# A Complex Interplay of Tandem- and Whole-Genome Duplication Drives Expansion of the L-Type Lectin Receptor Kinase Gene Family in the Brassicaceae

**DOI:** 10.1093/gbe/evv020

**Published:** 2015-01-28

**Authors:** Johannes A. Hofberger, David L. Nsibo, Francine Govers, Klaas Bouwmeester, M. Eric Schranz

**Affiliations:** ^1^Biosystematics Group, Wageningen University, The Netherlands; ^2^Chinese Academy of Sciences/Max Planck Partner Institute for Computational Biology, Shanghai, People's Republic of China; ^3^Laboratory of Phytopathology, Wageningen University, The Netherlands; ^4^Plant-Microbe Interactions, Department of Biology, Faculty of Science, Utrecht University, The Netherlands

**Keywords:** comparative genomics, polyploidy, gene duplication, Brassicaceae, L-type lectin receptor kinases, plant innate immunity

## Abstract

The comparative analysis of plant gene families in a phylogenetic framework has greatly accelerated due to advances in next generation sequencing. In this study, we provide an evolutionary analysis of the L-type lectin receptor kinase and L-type lectin domain proteins (L-type LecRKs and LLPs) that are considered as components in plant immunity, in the plant family Brassicaceae and related outgroups. We combine several lines of evidence provided by sequence homology, HMM-driven protein domain annotation, phylogenetic analysis, and gene synteny for large-scale identification of L-type *LecRK* and *LLP* genes within nine core-eudicot genomes. We show that both polyploidy and local duplication events (tandem duplication and gene transposition duplication) have played a major role in L-type *LecRK* and *LLP* gene family expansion in the Brassicaceae. We also find significant differences in rates of molecular evolution based on the mode of duplication. Additionally, we show that *LLP*s share a common evolutionary origin with L-type *LecRK*s and provide a consistent gene family nomenclature. Finally, we demonstrate that the largest and most diverse L-type *LecRK* clades are lineage-specific. Our evolutionary analyses of these plant immune components provide a framework to support future plant resistance breeding.

## Introduction

During plant evolution, individual genes and gene families have undergone selection for copy number through duplications, transpositions, and/or deletions. Such events can be detected by screening for patterns of syntenic or collinear genes (Coghlan et al. 2005; Woodhouse et al. 2011). Gene duplication and subsequent gene retention or loss (fractionation) are often attributed to recent and/or ancient whole-genome polyploidy events, for example at the origin of seed plants and angiosperms (Jiao et al. 2011). Whole-genome duplications (WGDs) can act as mechanism to buffer gene functions due to increased genetic redundancy and hence provide an important source of sub- or neofunctionalization driving genetic innovation (Chapman et al. 2006; Fawcett et al. 2009). For example, ohnolog genes (paralogous genes derived specifically from a WGD) encoding structurally similar enzymes have been shown to evolve toward extended substrate specificities or catalysis of novel reactions, whereas its ancestral gene retains its designated function (Roth et al. 2007). Similarly, distant genomic locations of ohnologs can lead to differential gene expression (Casneuf et al. 2006). Hence, it has been hypothesized that WGDs contributed to species diversity by driving trait evolution (Schranz et al. 2012). In this context, several studies highlight the contribution of WGD to the observed diversity across lineages as well as to extended gene function in a variety of organisms, including mammals (Gallardo et al. 1999), amphibians (Ptacek et al. 1994; Comber and Smith 2004), and plants (Wendel 2000; Osborn et al. 2003; Tate et al. 2005). Large-scale synteny is not observed for paralogs derived from small-scale events, such as tandem duplication (TD) and gene transposition duplication (GTD).

The Brassicaceae, also known as the mustard family, has many advantages to study and understand the contributions of whole-genome and gene duplications on plant genome evolution. It comprises several species for which well-assembled genomes are available, including *Arabidopsis thaliana, Arabidopsis lyrata*, *Brassica rapa*, *Thellungiella halophila,* and *Aethionema arabicum* (Swarbreck et al. 2008; Taji et al. 2008; Couvreur et al. 2010; Hu et al. 2011; Wang et al. 2011; Haudry et al. 2013). Analysis of many of these genome assemblies has provided insights into patterns of gene evolution, retention, and functionality (Thomas et al. 2006). Within the Brassicaceae family at least five polyploidy events can be detected that have occurred in the *A. thaliana* lineage, three of which have been studied extensively (Bowers et al. 2003; Van de Peer et al. 2009). This includes the “At-γ” event that occurred approximately 111 Myr before the lineage split of *A. thaliana* and the common grape vine *Vitis vinifera* (Wang et al. 2009) and which is shared by all eudicots (Jaillon et al. 2007; Vekemans et al. 2012). The less ancient “At-β” event occurred approximately 72 Myr after the split of the *Carica papaya* and *A. thaliana* lineages (Ming et al. 2008), and is restricted to the order Brassicales (Woodhouse et al. 2011). The most recent polyploidy event termed “At-α” occurred after the split of Brassicaceae and Cleomaceae approximately 40 Ma, and was followed by a lineage separation of *A. thaliana* and *A. lyrata* approximately 10 Ma (Bowers et al. 2003; Hu et al. 2011; Schranz et al. 2012). In addition to WGD events in plant genomes, local duplication events such as TD and GTD contributed to gene copy number variation and are currently the best understood drivers of gene retention and cluster expansion. TD is a result of single unequal crossing over (UCO) events, and/or multiple repeats thereof during DNA repair. UCO produces tandem duplicate genes organized in tandem-arrayed genes (TAR genes) that individually cluster with up to ten intervening genes (Kane et al. 2010). This leads to copy number variation in many plant gene families including several involved in plant disease resistance and glucosinolate biosynthesis (Parniske et al. 1999; Kliebenstein et al. 2001; Leister 2004; Hofberger et al. 2013). Interestingly, UCO can result in gene copies positioned in a head-to-tail direct orientation (Meyers et al. 2003). Alternatively, TD can also result from intrachromosomal rearrangements between direct and indirect repeats, producing gene copies with opposite head-to-head orientation within a tandem array. Note that depending on the orientation of adjacent tandem duplicates, common promoters can be shared. For example, intrachromosomal rearrangements caused the formation of the *A. thaliana* gene array *RRS1* and *RPS4* that function as a dual resistance gene system in defense against bacterial and fungal plant pathogens (Narusaka et al. 2009). Similarly, TD has significantly influenced the divergence of many disease resistance genes (i.e., *NB-LRR*s) that confer race-specific resistance in Brassicaceae and Solanaceae (Parniske et al. 1997; Leister 2004; Hofberger et al. 2014). In contrast to TD, GTD results in gene relocation to distant genomic positions and hence induces gene family dispersion across the entire genome. GTD copies transpose from ancestral to novel positions with the ancestral loci having fewer insertions and deletions (InDels) with shorter maximum InDel lengths. In addition, ancestral GTD “seed” loci have longer coding-regions and exon lengths than the novel copies (Wang et al. 2013). Overall, TD and GTD have been reported to frequently occur in diversified high-copy number gene families, such as those comprising *NB-LRR* disease resistance, Type I MADS-box transcription factor, F-Box, and B3 gene families (Freeling et al. 2008; Freeling 2009).

In plants, the perception of extracellular stimuli and subsequent signal transduction is often mediated by receptor-like kinases (RLKs), which can be divided into various subfamilies based on their extracellular domains (Shiu and Bleecker 2001a, 2001b). Plant RLKs underwent a dramatic expansion in comparison to those of other organisms with at least 610 and 1,100 members in *A. thaliana* and rice, respectively (Shiu and Bleecker 2003; Shiu et al. 2004) indicating their importance during plant adaptation. Several RLKs have been shown to play pivotal roles as pattern recognition receptors to mediate basal defense. Among these are the lectin receptor kinases (LecRKs), which are membrane-spanning receptors that contain an extracellular lectin domain and an intracellular Ser/Thr kinase domain (Bouwmeester and Govers 2009; Singh and Zimmerli 2013). LecRKs can be further subdivided based on their lectin domain composition into three categories; that is, the G-, C-, and L-type LecRKs (Bouwmeester and Govers 2009; Lannoo and Van Damme 2014). The G-type LecRKs, also known as S-domain RLKs, comprise functions in both plant self-incompatibility and defense (Kusaba et al. 2001; Gilardoni et al. 2011; Cheng et al. 2013). C-type LecRKs are named after their extracellular calcium-dependent lectin domain. This domain is commonly found in a plethora of innate immune receptors in mammalians (Geijtenbeek and Gringhuis 2009), but is rare in plants. The function of the C-type LecRKs remains thus far enigmatic. The third category consists of the L-type LecRKs which contain an extracellular legume-like lectin domain. L-type LecRKs are ubiquitous in plants, and have been identified in a variety of plant species, for example, cotton, cucumber, and rice (Vaid et al. 2012; Phillips et al. 2013; Wu et al. 2014). *Arabidopsis thaliana* was shown to contain 45 L-type LecRKs, which could be divided into nine distinct clades and seven additional so-called singletons that group distantly (termed “ambiguous” hereafter) (Bouwmeester and Govers 2009).

Recently, evidence has accumulated pointing toward roles of L-type LecRKs in biotic stress responses (Bouwmeester et al. 2011; Desclos-Theveniau et al. 2012; Singh et al. 2012; Wang et al. 2014). LecRK-I.9, for example, was identified as a *Phytophthora* resistance component (Bouwmeester et al. 2011), whereas LecRK-V.5 is involved in susceptibility to bacterial pathogens (Desclos-Theveniau et al. 2012). In addition, Singh et al. (2012) showed that LecRK-VI.2 is critical in defense in *A. thaliana* against both hemibiotrophic and necrotrophic phytopathogenic bacteria. L-type LecRKs also function in insect resistance, for example, *A. thaliana* LecRK-I.8 was shown to play a crucial role in defense triggered by egg-derived elicitors of the cabbage butterfly *Pieris brassicae* (Gouhier-Darimont et al. 2013). In addition, few L-type LecRKs are thus far described to function in response to abiotic stimuli and plant development (Wan et al. 2008; Deng et al. 2009; Xin et al. 2009; Wang et al. 2014).

Here, we employ several bioinformatics methods for the identification and comparison of L-type *LecRK* family members encoded in nine representative core-eudicot genomes. In a phylogenomics approach, we provide data to assess the differential impact of duplication modes driving L-type *LecRK* copy number expansion observed across the plant family Brassicaceae.

## Materials and Methods

### Plant Genome Annotations

Genome annotations for five Brassicaceae species: that is, *Ae. arabicum* v0.2 (Haudry et al. 2013), *A. thaliana* TAIR10 (Swarbreck et al. 2008), *A. lyrata* v1.07 (Hu et al. 2011), *B. rapa* (Wang et al. 2011a), and *Th. halophila* v1 (Taji et al. 2008); one Cleomaceae species: *Ta. hasslerania* v4 (Cheng et al. 2013); one Caricaceae species: *C. papaya* v0.5 (Ming et al. 2008); one Malvaceae species: *T. cacao* v1 (Argout et al. 2011); and one Vitaceae species: *V. vinifera* v2 (Jaillon et al. 2007) were obtained from Phytozome v9.1 (http://phytozome.org, last accessed December 12, 2014) (Goodstein et al. 2012).

### Reannotation of L-type *LecRKs* and *LLPs*

Protein and gene sequences of *A. thaliana* L-type LecRKs and LLPs were obtained using the Arabidopsis Information Resource website (TAIR10, http://www.arabidopsis.org, last accessed December 12, 2014). Possible pseudogenization of *A. thaliana* L-type *LecRK*s and *LLP*s was analyzed using available ATH1 microarray data sets at TAIR (data not shown). To identify orthologous L-type *LecRK*s and *LLP*s across the nine plant genomes, the Reciprocal Best Blast Hits (RBH) were determined using both *A. thaliana* gene and protein sequences as queries against the remaining eight plant genomes using NCBI (National Center for Biotechnology Information) BLAST 2.2.28+ (http://www.ncbi.nlm.nih.gov/news/04-05-2013-blast-2-2-28, last accessed December 12, 2014) (Altschul et al. 1990; Camacho et al. 2013) with an *e* value threshold of 1e-10. A total of three RBH sets (i.e., a length filtered protein pair set; a nonlength filtered protein pair set, and a nonlength coding sequence pair set with a size-filter threshold of 0.5-to-2 gene lengths) were retrieved after BLAST as previously described (Hofberger et al. 2014).

### Ohnolog Identification and Analysis

Ohnologs (collinear or syntenic copies of genes) of all putative L-type *LecRK* orthologs were identified through analysis of gene collinearity within and between all genomes using the “SynMap” algorithm within the CoGe package for comparative genomics (https://genomevolution.org, last accessed December 12, 2014) (Lyons et al. 2008). First, genes of each analyzed species that share syntenic orthologs to the *A. thaliana* L-type *LecRK*s and *LLP*s were determined by making use of DAGchainer (Haas et al. 2004) and quota align algorithms (Tang et al. 2011) within the CoGe package for comparative genomics (https://genomevolution.org/CoGe/GEvo.pl, last accessed December 12, 2014). The following parameter settings were used: Merging neighboring syntenic blocks, maximum distance between two blocks fixed at 350 genes; synonymous substitutions rates (Ks) with an average of 1.7 determined using CoDeML of the PAML package (Yang 2007) implemented in SynMap; five collinear genes to seed a syntenic block; and maximum of 20 nonsyntenic genes between syntenic genes to interrupt genomic blocks as previously described (Tang et al. 2011; Woodhouse et al. 2011). Second, within-species ohnologs (i.e., paralogs due to polyploidy) were determined by querying the target genomes against themselves. Microsynteny analysis within and between genomes was performed with GEvo (https://genomevolution.org/CoGe/GEvo.pl, last accessed December 12, 2014). The obtained syntenic gene set output was thereafter cleaned using a retention maximum of three ohnologs for each of the analyzed species.

### Anchor Paralog Identification and Protein Domain Prediction

Ortholog and ohnolog gene sets were combined to create a pool of homologous “anchor” genes. These gene sets of the analyzed target genomes were queried against the *A. thaliana* genome with a maximum target sequence threshold of 1. Each query sequence that aligned to an *A. thaliana* L-type *LecRK* or *LLP,* but not belonging to the “anchor” gene set, was defined as an anchor paralog. With the above-mentioned steps a complete set of L-type LecRK and LLP-encoding homologs present in every analyzed target species (orthologs, paralogs, and ohnologs) was created. As this approach may lead to false positives due to alignment of highly conserved linker sequence pairs, an additional filtering step was applied based on HMM-driven protein domain annotation using the iprscan_urlib.py script (https://www.ebi.ac.uk/Tools/webservices/download_clients/python/urllib/iprscan_urllib2.py, last accessed December 12, 2014) querying the EMBL server (http://smart.embl-heidelberg.de, last accessed December 12, 2014) (Letunic et al. 2012). Protein motifs were determined using InterProScan 4 (http://www.ebi.ac.uk/Tools/pfa/iprscan, last accessed December 12, 2014) (Apweiler et al. 2001; Hunter et al. 2009) and the bioinformatics tools SMART, Superfamily, ProDom, PRINTS, PROSITE, PIR, Pfam, TIGRFAMs, PANTHER, Profile, Gene3D, HAMAP, TMHMM, and SignalP.

### Identification of Mode of Gene Duplication

*Arabidopsis thaliana* L-type *LecRK* ohnolog gene copies were obtained based on the blocks described by Bowers et al. (2003) and updated according to Thomas et al. (2006). Determination of ohnolog duplicates in all other genomes was utilized using the “SynMap” algorithm integrated into CoGe with above-described preferences. Tandem duplicate genes were obtained using BLASTP hits within a maximum of ten consecutive intervening gene spacers as previously described (Rizzon et al. 2006). To identify gene transposition duplicate (GTD) partners among homolog genes, all non-tandem non-ohnolog duplicate target sequences were queried against the whole set of target genes using BLASTP with an *e* value threshold of 1e-30. Closest homologs were scored as GTD partners. Putative transpositions were confirmed using the gene transpositional database (Freeling et al. 2008). Duplicated gene copies belonging to tandem-duplicated ohnologs (TD-α genes) by sharing similar evolutionary patterns with tandem duplicates were obtained and confirmed using the methods described by Wang et al. (2013). Statistical significance of retained ohnolog fractions among target genes compared with the background of genome-wide ohnolog fractions was determined using a Fisher’s exact test on count data integrated to the R package for statistical computing (http://www.r-project.org, last accessed December 12, 2014).

### Coding Sequence Alignment and Determination of Ka/Ks-Values to Assess Divergence

Coding sequence alignments of homologous genes were compiled in Mesquite (Maddison W and Maddison D 2010) and manually cleaned to remove premature stop codons and gaps. Other alignments were generated using Prank (http://www.ebi.ac.uk/goldman-srv/webprank, last accessed December 12, 2014) (Loytynoja and Goldman 2010) with default settings. Ka/Ks was calculated using the KaKs calculator (https://code.google.com/p/kaks-calculator/wiki/KaKs_Calculator, last accessed December 12, 2014) (Zhang et al. 2006). Average divergence rates between respective tandem, ohnologs, gene transposition duplicates, and tandem–ohnolog homologous sequences were computed as previously described (Hofberger et al. 2014).

### Sequence Annotation and Alignment

Alignments of full length protein sequences were compiled using Mesquite version 2.74 (Maddison W and Maddison D 2010). Removal of stop codons and sequence trimming was performed as previously described (Hofberger et al. 2014). Sequence alignment was performed using Prank relying on default settings (http://www.ebi.ac.uk/goldman-srv/webprank, last accessed December 12, 2014) (Loytynoja and Goldman 2010).

### Phylogenetic Analysis

Maximum-likelihood phylogenetic trees were constructed with full-length protein sequences using the RAxML web-server at the CIPRES portal (http://sco.h-its.org/exelixis/web/software/raxml/index.html; last accessed December 12, 2014) (Stamatakis 2014). Maximum-likelihood searches and estimate proportion of invariable sites were selected as parameters. The robustness of the phylogenetic trees was assessed by performing bootstrap resampling using 100 replicates. All phylogenetic trees were rooted with protein sequences of WAK1 (AT1G21250), PERK1 (AT3G24550), the C-type LecRK AT1G52310, and the G-type LecRKs ARK1 (AT1G65790) and CES101 (AT3G16030). MrBayes version 3.2.2 (http://www.phylo.org/portal2/oldmrbayeshybrid_tg!input.action, last accessed December 12, 2014) (Ronquist et al. 2012) was used to generate Bayesian trees using the following parameters: Rates allowed to vary among four gamma categories; nucleotide state frequencies mixed (Dirichlet model); a uniform gamma shape parameter allowed to vary between 0 and 200 analysis to run for 50 million generations; each generation consisting of two independent runs for four chains each, one of which was heated at a temperature of 0.2 to keep the heated chain in motion; samples were taken every 5,000 generations; and burn-in time was set at 12,500,000 samples. Bayesian inference trees were constructed by using CIPRES (http://www.phylo.org/sub_sections/portal, last accessed December 12, 2014) (Miller et al. 2009). Convergence of the parameters and model likelihood between runs were checked in Tracer version 1.5 (http://beast.bio.ed.ac.uk/Tracer, last accessed December 12, 2014) after which .p- and .t-files were combined as previously described (Rambaut and Drummond 2009). Con files (.con) were generated in MrBayes and contained the Bayesian 50%-majority rule consensus trees. FigTree software was used to generate and edit the phylogenetic trees (http://tree.bio.ed.ac.uk/software/figtree, last accessed December 12, 2014). Results were scored positive once the effective sampling size of all parameters was above 100. Tree branches supported by posterior probabilities (PP) below 0.7 were considered as weak and above 9.0 as strong.

## Results

### Curation of *A. thaliana* L-type *LecRK*s and *LLP*s

**Fig. 1.—:**
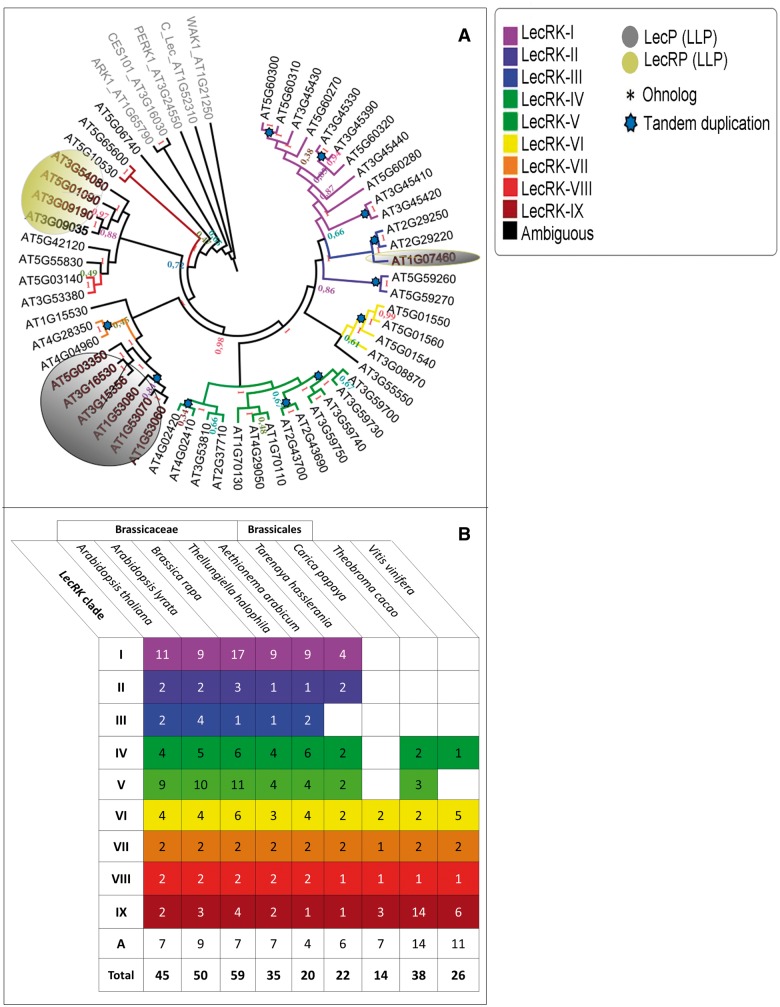
Phylogeny and classification of *A. thaliana* L-type LecRKs and LLPs. (*A*) Phylogeny of 43 full-length L-type LecRKs and 11 LLPs in *A. thaliana*. We identified two LLP clades; LecPs (lacking transmembrane domains) and LecRPs (with transmembrane domains) which are highlighted in dark gray and ochre, respectively. Color-coding was adopted according to Bouwmeester and Govers (2009). TD events are indicated by light blue stars. The tree was rooted using the *A. thaliana* G-type LecRKs CES101 and ARK1, the C-type LecRK AT1G52310, and the Wall-associated kinases WAK1 and PERK1. Clade-support bootstrap values range from 0.80 to 0.94. (*B*) Clade assignment of 309 *LecRK*s identified across nine analyzed genome annotations. Colors represent the nine clades originally described by Bouwmeester and Govers (2009). “A” refers to ambiguous genes (singletons).

**Fig. 2.—:**
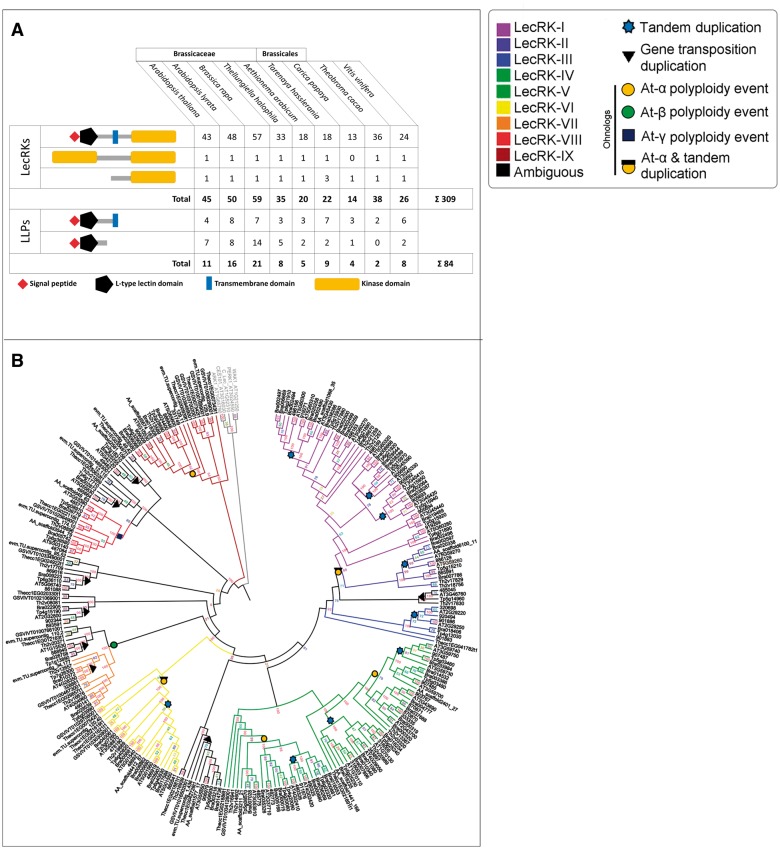
Classification of L-type LecRKs and LLPs identified in nine plant species. (*A*) Domain composition of 309 L-type LecRKs and 84 LLPs across Brassicaceae, Brassicales, *T. cacao,* and *V. vinifera*. L-type LecRKs containing two kinase domains are present in all analyzed species except *C. papaya.* Note that *T. cacao* lacks LecPs. (*B*) Cladogram based on the legume-like lectin domains of Brassicaceae L-type LecRKs from *A. thaliana*, *A. lyrata*, *B. rapa, Th. halophila,* and *Ae. arabicum.* Further included are 63 legume-like lectin domain sequences from four other families: *Ta. hasslerania* (Cleomaceae), *T. cacao* (Malvaceae), *C. papaya* (Caricaceae), and *V. vinifera* (Vitaceae) with support values indicated on key nodes. Number-only IDs refer to expressed genes present in the “Araly1”-annotation (*A. lyrata*). The phylogenetic tree was rooted with the extracellular domains of the G-type LecRKs CES101 and ARK1, the C-type LecRK AT1G52310, and the Wall-associated kinases WAK1 and PERK1 as outgroup sequences. Clade support bootstrap values range from 0.70 to 0.95. For all species, the L-type LecRKs cluster to nine distinct clades (colored) corresponding to the clade assignment of the *A. thaliana* L-type LecRKs including those without clear affiliation to a distinct clade (ambiguous). Symbols placed on nodes represent the different duplication modes: that is, At-α WGD event (orange circles), At-α ohnologs subjected to TD (TD-α genes) (orange circle with black square), TD event (light blue stars), gene transposition duplicates (black triangle), and more ancient polyploidy events: At-β (blue square) and At-γ (green circles). Symbols mark last common duplication events. Six of nine clades are specific to Brassicaceae, Cleomaceae and Caricaceae, whereas the rest of the clades are shared between Brassicales and Vitales. Ambiguous LecRKs are spread across the tree and across the families.

In a first step, we compiled a list of the 45 L-type *LecRK*s that have previously been described in *A. thaliana* (supplementary table S1, Supplementary Material online) (Bouwmeester and Govers 2009). Phylogenetic analysis placed 37 L-type *LecRK*s into nine distinct clades and identified seven singleton genes (e.g., no clear relationship to one distinct clade or “ambiguous” genes). We confirmed these previous results with our phylogenetic analysis (fig. 1*A* and *B*). Furthermore, we included the ten *LLP* genes previously identified by Armijo et al. (2013) that encode so-called Legume-like lectin proteins. LLPs contain a legume-like lectin domain but lack a kinase domain. In addition, we found another *LLP*; bringing the total count of *A. thaliana LLP*s to 11 (table 1). In summary, the *A. thaliana* genome encodes 56 proteins containing a putative legume-like lectin domain (IPR001220) (fig. 2*A*). For the 11 *LLP*s, we propose a uniform gene nomenclature based on their phylogenetic relationship (fig. 1*A*). LLPs form two strongly supported monophyletic clades, one consisting of six and the other of four members. The remaining one is an ambiguous LLP because it groups distantly. In line with the nomenclature proposed by Bouwmeester and Govers (2009), the *LLP* clades were named using Roman numerals. The largest clade of six comprises LLPs that lack a transmembrane domain, for which we propose the term L-type lectin proteins (Clade I: LecPs). In contrast, the other LLP members share in addition to the legume-like lectin domain a transmembrane domain, and are herewith proposed to be named L-type lectin receptor proteins (Clade II: LecRPs) (table 1, fig. 2*A*). Interestingly, our phylogenetic analysis shows that LecP-I.1 (At1g07460) groups with the LecRK-III clade (fig. 1*A*), whereas all other LecPs show a shared sequence similarity with L-type LecRKs belonging to clade VII (fig. 1*A*), and this indicates that LLPs share independent evolutionary histories with L-type LecRKs.

**Table 1:** Classification of *LLP* Loci in *A. thaliana* Including Information on Encoded Proteins

Gene Information						Protein Information				
Proposed LLP Clade Classification	Proposed Gene Name	Locus	Tandem Duplicate?	Length (bp)	Uniprot Accession	Length (AA)	Signal Peptide	No. of TM Motifs	Domain Configuration	Reference
LecRP	*LecRP-I.1*	AT3G09035	yes	1017	Q3EBA4	338	yes	1	L-type lectin-TM	Armijo et al. (2013)
	*LecRP-I.3*	AT3G09190	yes	1038	Q9SS71	345	yes	1	L-type lectin-TM	This manuscript
	*LecRP-S.1*	AT3G54080	no	1053	Q9M395	350	yes	1	L-type lectin-TM	Armijo et al. (2013)
	*LecRP-I.2*	AT5G01090	no	1062	Q9LFC7	353	yes	1	L-type lectin-TM	Armijo et al. (2013)
LecP	*LecP-I.1*	AT1G07460	no	777	Q4PT39	258	no	0	L-type Lectin	Armijo et al. (2013)
	*LecP-I.2*	AT1G53060	yes	729	Q9LNN1	242	no	0	L-type Lectin	Armijo et al. (2013)
	*LecP-I.3*	AT1G53070	yes	819	Q9LNN2	272	yes	0	L-type Lectin	Armijo et al. (2013)
	*LecP-I.4*	AT1G53080	yes	852	Q9LNN3	283	yes	0	L-type Lectin	Armijo et al. (2013)
	*LecP-I.5*	AT3G15356	no	816	Q9LJR2	271	yes	0	L-type Lectin	Armijo et al. (2013)
	*LecP-I.6*	AT3G16530	no	831	Q9LK72	276	yes	0	L-type Lectin	Armijo et al. (2013)
	*LecP-I.7*^a^	AT5G03350	no	825	Q9LZF5	274	yes	0	L-type Lectin	Armijo et al. (2013)

^a^Alias SAI-LLP1 (Armijo et al. 2013).

### Duplication Analysis of *A. thaliana* L-type *LecRK*s and *LLP*s

To establish the relationship between gene duplication and genetic divergence, the chromosomal locations of the L-type *LecRK*s were confirmed using the *A. thaliana* locus codes. Results show that the L-type *LecRK*s are organized in nine gene clusters distributed over the five *A. thaliana* chromosomes, with the highest density on chromosome V followed by chromosome III (Bouwmeester and Govers 2009). All L-type *LecRK*s are located to regions covered by ohnolog blocks due to the most recent ancient polyploidy event. Furthermore, we localized the two *A. thaliana* clusters possessing the highest target gene density at two independent tandem duplicate supergene clusters on chromosomes III and V. *Arabidopsis thaliana* clade V L-type *LecRK*s are located in proximity on chromosomes I–III (supplementary table S1, Supplementary Material online). Notably, one large tandem array containing *LecRK-V.5* (At3g59700), *LecRK-V.6* (At3g59730), *LecRK-V.7* (At3g59740), and *LecRK-V.8* (At3g59750) was found to be specific for *A. thaliana* as orthologs in all other species were singletons (array 14 in supplementary table S1, Supplementary Material online). Likewise, we investigated the genomic locations of the *A. thaliana LLP*s. These were predominantly located on chromosomes III and V, of which several cluster together with L-type *LecRK*s. Among these are *LecRP-I.1* (At3g09035) and *LecRP-I.3* (At3g09190) that share chromosomal location with *LecRK-VI.1* (At3g08870). Moreover, *LecRP-I.2* (At5g01090) and *LecRK-VI.2* (At5g01540) are located in each other chromosomal proximity (supplementary table S1, Supplementary Material online). Again, this shows that that *LLP*s share an evolutionary history with L-type *LecRK*s. In this context, the observed degree of sequence similarity and domain conservation may be due to ancient sub- and neofunctionalization following gene- and genome duplication.

### Domain Conservation and Ortholog Retention across the Brassicaceae

As a next step, a combination of *A. thaliana* L-type *LecRK* orthologs was obtained for eight genome assemblies by RBH analysis (supplementary table S2, Supplementary Material online). Likewise, L-type *LecRK* ohnologs were curated for all analyzed genomes (supplementary table S3, Supplementary Material online). Both data sets were merged to create a pool of “anchor” genes for every analyzed genome annotation. This pool of putative “anchor” genes was used in an additional BLAST analysis against the various genomes to screen for target gene paralogs. This additional screen was necessary because it became evident that ortholog assignment based on RBH only misses many true orthologs in lineages with duplicate-rich genomes (Dalquen and Dessimoz 2013). In this way, we identified a total of 393 genes encoding a legume-like lectin domain, of which 309 are L-type *LecRK*s (fig. 2*A*). In line with the phylogenetic relationship of the *A. thaliana* L-type *LecRK*s, all Brassicaceae contain the nine clades of L-type *LecRK*s and at least four ambiguous gene family members that encode proteins with the conserved L-type LecRK domain composition (fig. 2). However, species-specific differences apply with increased phylogenetic distance. For example, *Ta. hassleriana* of the Cleomaceae is a closely related sister lineage to all mustard family members but its genome annotation does not contain clade III orthologs. The more distant species *T. cacao* lacks L-type LecRKs aligning to clades I–III, and *C. papaya* lacks target genes from clades I–V, as well as the orthologs of the two “ambiguous” target genes At2g32800 (*LecRK-S.2*) and At3g46760 (*LecRK-S.3*). The common grape vine *V. vinifera*, the most distant Brassicaceae outgroup analyzed in this study, lacks orthologs grouping to L-type *LecRK* clades I, II, III, and V.

### L-type *LecRK* Orthologs and Ohnologs across the Brassicaceae and Several Outgroups

When investigating the genomic context of orthologous target gene pairs, we found that all analyzed genomes retained a fraction of the respective orthologs within a given syntenic region (i.e., are syntenic to *A. thaliana* L-type *LecRK* orthologs or ohnologs) (supplementary tables S2 and S3, Supplementary Material online). Notably, the closest related sister lineage *A. lyrata* has ohnologs to 39 *A. thaliana* L-type *LecRK*s, corresponding to a retention score of 87% (supplementary table S3, Supplementary Material online). This score decreases with increased phylogenetic distance of Brassicaceae lineages, as indicated by the values for the crop species *B. rapa* (78%), the saltwater cress *Th. halophila* (62%), the early-diverged mustard *Ae. arabicum* (69%), and the closest mustard outgoup *Ta. hasslerania* (36%), as well as the more diverged crop species *C. papaya* (18%), *T. cacao* (29%) and *V. vinifera* (16%). These results are consistent with previous studies reporting an erosion of synteny across lineages relative to their phylogenetic distance (Lyons et al. 2008; Hofberger et al. 2014). In addition, we investigated the retention of *LLP*s in the various Brassicaceae species. This revealed that between 91% (*A. lyrata*) and 45% (*Ae. arabicum*) of all *LLP*s identified in *A. thaliana* are retained within the analyzed Brassicaceae species. Interestingly, the Brassicaceae outgroup *Ta. hasslerania* retained a higher fraction of *LLP* orthologs (73%) than the basal Brassicaceae *Ae. arabicum*, and this is consistent with the species-specific genome triplication event evident for this Cleomaceae species (Cheng et al. 2013). In contrast, all other Brassicales as well as *V. vinifera* only contain one *LLP* gene which is orthologous to *A. thaliana LecRP-I.2* (AT5G01090), corresponding to a retention score of 10% (supplementary tables S4 and S5, Supplementary Material online).

### Different Modes of Duplication Affect L-type *LecRK* and *LLP* Copy Number Variation

In a next step, we identified both TD and WGD events that have influenced copy number variation and molecular evolution of the L-type *LecRK* and *LLP* gene families across all analyzed genomes. For *A. thaliana* L-type *LecRK*s and *LLP*s, we scored both tandem- and ohnolog duplicates based on previously published definitions (see Materials and Methods) (supplementary table S6, Supplementary Material online, and table 2). The obtained results revealed that a relatively large fraction of ohnologs (37%) was retained from ancient polyploidy events among all identified L-type *LecRK* and *LLP* genes within all genomes. Compared with the average of genome-wide ohnolog fractions across all genomes (i.e., 30%), this indicates a significant overretention of whole-genome duplicates among L-type *LecRK*s and *LLP*s (table 3). Note that species-specific differences apply. For example, *B. rapa* and *Ta. hasslerania*, which both underwent a lineage-specific genome triplication event, show higher fractions of genome-wide ohnologs compared with the other lineages (53% and 48%, respectively, compared with 22% for *A. thaliana*). In summary, statistical analysis based on a Fisher’s exact test revealed a significant enrichment of ohnologs among genes encoding a legume-like lectin domain for five of the nine genomes that we investigated (table 3). Likewise, we identified a 55% fraction of genes in tandem arrays among all identified L-type *LecRK*s and *LLP*s (table 4). All identified tandem duplicate genes group to a sum of 54 distant tandem arrays distributed across all analyzed genomes (supplementary table S1, Supplementary Material online), with an average of 2.9 genes per tandem array and 5.9 genes in the largest identified tandem array (table 4). Again, differences were detected in the species-wise tandem duplicate fractions among target genes, varying from 29% in *A. lyrata* to 68% in *T. cacao* (table 4). We could, however, not detect any tandem duplicate genes in *Ae. arabicum*. This could be due to the fact that we have used a draft version of the *Ae. arabicum* genome annotation that is based on large-scale integration of RNAseq data. This draft version may include mis-annotations of small open reading frames with fusions of tandem duplicates due to similar transcripts. The number of independent clusters of tandem duplicates was found to vary across species; that is from 11 distant tandem arrays in the Brassicaceae species *B. rapa* (that underwent a species-specific genome triplication) to one tandem array only in the Brassicales crop *C. papaya*. The more distant Brassicales crop *T. cacao* contains the highest average number of genes per array, whereas tandem arrays in the most distant analyzed outgroup *V. vinifera* are lowest in gene count across all analyzed species. Assessment of gene count within the largest array present in all analyzed species revealed maximums of ten for *B. rapa* and minimums of three in *C. papaya* and *V. vinifera* (table 4).

**Table 2:** Duplicate *LLP* Gene Pairs in *Arabidopsis thaliana* and Mode of Duplication

Duplicate 1		Duplicate 2		
AGI	Name	AGI	Name	Duplication Mode
AT1G53060	*LecP-I.2*	AT1G53070	*LecP-I.3*	TD
AT1G53070	*LecP-I.3*	AT1G53080	*LecP-I.4*	TD
AT1G53080	*LecP-I.4*	AT1G53070	*LecP-I.3*	TD
AT3G09035	*LecRP-I.1*	AT3G09190	*LecRP-I.3*	GTD
AT3G09190	*LecRP-I.3*	AT3G09035	*LecRP-I.1*	GTD
AT3G15356	*LecP-I.5*	AT3G16530	*LecP-I.6*	GTD
AT3G16530	*LecP-I.6*	AT3G15356	*LecP-I.5*	GTD
AT5G03350	*LecP-I.7*	AT3G15356	*LecP-I.5*	GTD
AT3G54080	*LecRP-S.1*	AT5G01090	*LecRP-I.2*	Ohnolog
AT5G01090	*LecRP-I.2*	AT3G54080	*LecRP-S.1*	Ohnolog
AT1G07460	*LecP-I.1*	AT2G29220	*LecRK-III.1*	Tandem and ohnolog (TD-α)

**Table 3:** Ohnolog Duplicate Fractions among Genes Encoding a Protein with a L-type Lectin Domain

	Genome-Wide		Genes Encoding a L-type Lectin Domain				
Species	Number of Genes	Ohnolog Fraction (%)	*LecRK*s	*LLP*s	Sum	Ohnolog Fraction (%)	Enrichment^a^
*Arabidopsis thaliana*	27,416	22	45	11	56	29	Yes
*Arabidopsis lyrata*	32,670	28	50	16	66	35	Yes
*Brassica rapa*	40,367	53	59	21	80	40	No
*Thellungiella halophila*	25,191	32	35	8	43	40	Yes
*Aethionema arabicum*	22,230	29	20	5	25	56	Yes
*Tarenaya hasslerania*	31,580	48	22	9	31	48	No
*Carica papaya*	27,793	7	14	4	18	11	No
*Theobroma cacao*	29,452	32	38	2	40	33	No
*Vitis vinifera*	23,092	22	26	8	34	38	Yes
Σ/Average		30	309	84	393	37	Yes

^a^According to Fisher’s exact test (*P* < 0.01).

**Table 4:** Tandem Duplicate Fractions among Genes Encoding a Protein with a L-type Lectin Domain

Species	Genes Encoding an L-Type Lectin Domain	Number of Tandem Duplicates	Fraction of Tandem Duplicates (%)	Number of Tandem Arrays	Average Size of Arrays^a^	Number of Genes in Largest Array^a^
*Arabidopsis thaliana*	56	31	55	10	3.1	6
*Arabidopsis lyrata*	66	19	29	8	2.4	4
*Brassica rapa*	80	34	43	11	3.1	10
*Thellungiella halophila*	43	19	44	7	2.7	5
*Aethionema arabicum*^b^	25	0	0	0	0	0
*Tarenaya hasslerania*	31	10	32	4	2.5	4
*Carica papaya*	14	3	16	1	3.0	3
*Theobroma cacao*	40	27	68	8	3.4	7
*Vitis vinifera*	34	11	32	5	2.2	3
Σ/Average	389	154	40	54	2.9	4.57

^a^Tandem array refers to a locus containing one distinct cluster of tandemly arrayed genes.

^b^Early-build genome annotation.

**Fig. 3.—:**
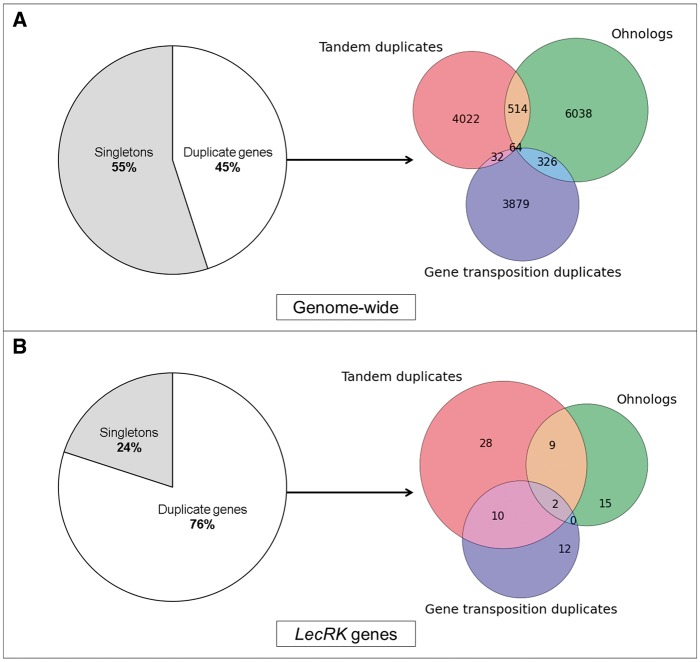
Venn-diagrams illustrating genome-wide average and L-type *LecRK* gene duplication fractions. Tandem duplicates (red), ohnolog duplicates (green), and gene transposition duplicates (blue). (*A*) Duplicates among all protein-coding genes present in the *A. thaliana* genome. (*B*) Duplicates among all L-type *LecRK*s present in the *A. thaliana* genome.

Previous reports indicated that tandem duplicate gene clusters are the birthplace of transposed duplicate copies (Freeling 2009; Wang et al. 2013). For the *A. thaliana* genome, a gene transpositional database has previously been made available (Freeling et al. 2008). These data facilitated scoring of GTD L-type *LecRK* copies. As a result, we referenced all L-type *LecRK* duplicates to either tandem-, ohnolog-, or gene transposition duplication modes and compared those with the observed genome-wide fractions of duplicate classes. Initially, we found that 45% of all protein-coding genes in the *A. thaliana* genome comprise duplicate genes (fig. 3*A*). As previously reported for all *A. thaliana* protein-coding genes (Hofberger et al. 2013), ohnolog copies comprise 22%, whereas copies due to TD or GTD comprise 15% and 14%, respectively (4,022/27,416 for TD and 3,879/27,416 for GTD) (fig. 3*B*). For the subset of L-type *LecRK* genes, we observed different trends in duplication. In *A. thaliana*, 34 of the 45 L-type *LecRK*s comprise duplicates, corresponding to a 76% fraction (fig. 3*C*). In total, we found that 26% of the L-type *LecRK*s in *A. thaliana* transposed at least once after the origin of the Brassicales (i.e., 12 of 45). Note that all L-type *LecRK* GTD copies are members of tandem duplicate gene clusters (fig. 3*D*). For the *LLP*s, the GTD fraction is 45%, that is 5 of the 11 *A. thaliana LLP*s (supplementary table S1, Supplementary Material online). Transposition times of most *A. thaliana* GTD copies have been estimated previously (Thomas et al. 2006; Freeling et al. 2008). Based on this, we estimated the transposition times for the transposed L-type *LecRK*s to the epoch of At-α (approximately 25–50 Ma) and even earlier polyploidy events, for example At-β (approximately 50–72 Ma) which are shared by Brassicales (Jiao et al. 2011; Woodhouse et al. 2011). Many other genes have been reported to have been expanded due to transposition duplication including *B3*, *LCR*, and *TRAF* genes that duplicated after *A. thaliana* diverged from *C. papaya* (Woodhouse et al. 2011). In this context, we uncovered a connection of GTD and other types of duplications with consequences for molecular evolution (see below).

Hereafter, we assessed the fractions of *A. thaliana* L-type *LecRK* and *LLP* ohnologs that have been subjected to TD following polyploidy, hereafter termed TD-α duplicates (table 2 and supplementary table S6, Supplementary Material online). This revealed a 20% fraction of TD-α duplicates among *A. thaliana* L-type *LecRK* genes (9 of 34 nonsingleton genes) (supplementary table S1, Supplementary Material online, and fig. 3*D*). This value is consistent with the 20% of TD-α duplicates found among the glucosinolate biosynthetic genes in *A. thaliana* (Hofberger et al. 2013) (see discussion). In contrast, none of the tandem duplicates among *LLP*s contains ohnologs that date back to the At-α WGD event (supplementary table S1, Supplementary Material online). Furthermore, our phylogenetic analysis revealed that TD-α genes are prone to clades I and V, and Brassicales-specific. These two clades are hence the most dynamic L-type *LecRK* clades among the analyzed plant species (fig. 1*A*). Here, we show that a 29% fraction of genes retained after ancient polyploidy events for the merged set of *A. thaliana LLP* and L-type *LecRK*s (table 3). Likewise, 55% of genes within this merged set comprise members of tandem arrays (table 4). Moreover, 30% of L-type *LecRK* and *LLP* genes transposed at least once after the origin of Brassicales, whereas the *A. thaliana* L-type *LecRK*s were found to belong to a GTD fraction of 26% (supplementary table S1, Supplementary Material online). In comparison to the genome-wide average, there is a significant difference in the proportions of tandem-, gene transposition-, and ohnolog duplicate fractions in L-type *LecRK*s (fig. 3). In addition, a clear impact of both TD and WGD (TD-α genes) was detected among the L-type *LecRK* genes.

### Molecular Evolution of L-Type *LecRKs* Is Impacted by Different Modes of Duplication

**Fig. 4.—:**
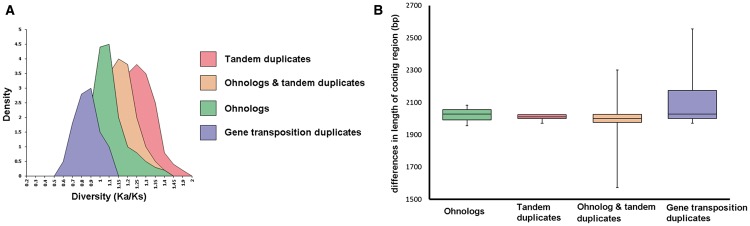
Analysis of divergence of L-type *LecRK*s based on mode of gene duplication in *A. thaliana*. (*A*) Molecular evolution rates of L-type *LecRK* gene pairs based on Ka/Ks values following TD (red), GTD (blue), divergence of ohnologs due to WGD (green), and divergence of ohnologs that have been subjected to TD (TD-α genes) (ochre). (*B*) Divergence of duplicate gene coding sequence length following the aforementioned duplication modes with identical color-coding.

Determination of synonymous substitution rates per synonymous sites (Ks) is a common procedure to determine the evolutionary age and divergence level of gene copies (Casneuf et al. 2006; Wang et al. 2013). In this context, comparing divergence rates provides insights into the differential impact of gene duplication modes (Casneuf et al. 2006; Bailey et al. 2009; Wang et al. 2011a). Hence, we calculated the Ka/Ks values of the L-type *LecRK*s that date back to different duplication modes in *A. thaliana*. We observed differential patterns of selection following all analyzed duplication modes (table 5, fig. 4*A*). Tandem duplicate L-type *LecRK*s show the highest average rates of molecular evolution (Ka/Ks = 1.23), indicating strong positive or Darwinian selection. Interestingly, lower rates of positive selection were determined for TD-α genes that comprise tandem duplicate ohnolog copies (Ka/Ks = 1.13) as well as ohnolog duplicate gene pairs (Ka/Ks = 1.11). Ka/Ks-values equal to 1 indicate neutral (or absence of) selection. L-type *LecRK* copies due to GTD showed the lowest rate of molecular evolution, that is, a Ka/Ks value of 0.94, implying moderate purifying (or stabilizing) selection (table 5, fig. 4*A*). The GTD duplicate class comprises mostly ambiguous L-type *LecRK*s and members of clades V and VII (supplementary table S6, Supplementary Material online).

**Table 5:** Molecular Evolution Rates Following Different Modes of *LecRK* Duplication

Duplication Mode	Ka	Ks	Ka/Ks
Gene transposition duplicates	2.6	2.78	0.94
Ohnolog duplicates	2.98	2.68	1.11
Tandem and ohnolog duplicates (TD-α genes)	2.58	2.29	1.13
Tandem duplicates	2.72	2.42	1.23

Note.—Ka, nonsynonymous substitutions per nonsynonymous site; Ks, synonymous substitutions per synonymous site.

Furthermore, we compared gene lengths of L-type *LecRK* copies due to different duplication events using gene-coding sequences (CDS). All CDS were compiled and clustered based on the duplication modes and the difference in coding-region lengths was estimated (fig. 4*B*). In this analysis, tandem duplicate gene copies display the lowest observed average both for coding-region length and variation thereof, whereas GTD copies display the highest. In contrast, coding-region length of TD-α duplicates displays the highest variation. These findings are consistent with previous studies, uncovering a connection between gene length and duplicate origin (Woodhouse et al. 2011).

## Discussion

As sessile organisms, plants are permanently exposed to a plethora of microbes, including plant pathogens. Hence, the perception of biotic stimuli is crucial for plant survival. The initial detection of these stress factors and subsequent induction of defense signaling is largely governed by RLKs. One class of RLKs considered to function as potential immune receptors are the L-type LecRKs, which comprise an extracellular legume-like lectin domain hypothesized to perceive nonself-associated molecules (Bouwmeester et al. 2011; Desclos-Theveniau et al. 2012; Singh et al. 2012; Wang et al. 2014). Also, the LLPs that similar to L-type LecRKs contain a legume-like lectin domain, but are lacking a kinase domain, have been suggested to play roles in plant defense (Armijo et al. 2013; Lannoo and Van Damme 2014).

In this study, we used bioinformatics techniques in a comparative genomics approach to elucidate the evolutionary history of the superfamily of legume-like lectin domain-encoding genes in Brassicaceae and related families. This methodology confirmed all previously identified L-type *LecRK*s (Bouwmeester and Govers 2009) and identified 11 *LLP* genes in *A. thaliana*; ten of which were described before by Armijo et al. (2013) (table 4). We revealed that 37% of all target genes identified across all species comprise ohnolog gene copies due to WGD events. Compared with the genome-wide averages of duplicates due to polyploidy in all analyzed species, we uncovered a significant enrichment for ohnologs among genes encoding a legume-like lectin domain (table 1). Investigating local duplication events, we scored tandem duplicate gene copies among L-type *LecRK*s and *LLP*s in all analyzed species and revealed that the majority of target genes localize to arrays of tandem duplicate genes in *A. thaliana* and *T. cacao* (55% and 68%, respectively). Including all other genome assemblies, a global 40% fraction of all identified L-type *LecRK*s and *LLP*s are organized in tandem arrays (table 2). Based on rates for molecular evolution (i.e., Ka/Ks values), we find that tandem duplicate *LecRK*s potentially have been subjected to stronger positive selection in comparison to copies resulting from other duplication modes, a characteristic that also has been described for *NB-LRR* resistance genes in Brassicaceae and Solanaceae (Hofberger et al. 2014). Overall, this indicates that the TD events drive divergence of L-type *LecRK* paralogs and orthologs and thereby could influence functional specialization in plant immunity.

Tandem arrays exist as a result of UCO (Kane et al. 2010), and result into duplicate genes positioned with a direct orientation. Nine L-type *LecRK* tandem arrays were detected to exhibit a head-to-tail orientation. The exception is the gene pair of *LecRK-V.7* and *LecRK-V.8*, which is positioned in a head-to-head orientation, indicating a potentially shared promoter region. This phenomenon is attributed to intrachromosomal recombination between direct and indirect repeats. These two L-type *LecRK*s fall under the fraction of tandem duplicate genes that exist as a result of intrachromosomal recombination in *A. thaliana* (Schuermann et al. 2005). Head-to-head orientation of tandem duplicates has been shown to be relevant for gene function. This includes genes involved in plant innate immunity, as previously shown for the *RRS1*/*RPS4* gene pair that encodes a dual *NB-LRR*-mediated resistance system (Narusaka et al. 2009). Further functional studies to elucidate the contribution of the spatial orientation of *LecRK-V.7* and *LecRK-V.8* in plant immunity are needed, especially as *LecRK-V7* seems to play a role in defense against *Phytophthora* pathogens and the bacterium *Pseudomonas syringae* (Wang et al. 2014). For the *A. thaliana* L-type *LecRK*s, our results further demonstrate a significantly increased fraction of gene copies due to a combination of WGD and TD (TD-α genes) compared with the genome-wide average (fig. 3). Interestingly, TD-α gene pairs evolve faster than ohnologs following duplication. The majority of TD-α L-type *LecRK*s groups to clades I and V (supplementary table S5, Supplementary Material online). Note that the largest L-type *LecRK* tandem array in *A. thaliana* contains ohnolog copies also while grouping to an underfractionated homologous genomic region (supplementary table S1, Supplementary Material online). Hence, expansion of gene copy number within the L-type *LecRK* clades I and V is largely due to a combination of whole genome- and duplication (TD-α duplication), indicating that their evolution is more dynamic compared with other L-type *LecRK* clades. We hypothesize that the underlying increased copy number occurred at the time of the At-α polyploidy event after the Brassicaceae and Cleomaceae lineage split (Schranz and Mitchell-Olds 2006; Couvreur et al. 2010). This phenomenon was also reported among glucosinolate biosynthetic genes, which show a 20% fraction of genes due to TD-α duplication (Hofberger et al. 2013). Also, recent WGD and TD seem to have greatly influenced the expansion and retention of L-type *LecRK*s in clades I, II, III, and V among core Brassicales, which might be related to the increased degree of functional divergence observed for target genes in this family. More ancient WGD events also had an impact on L-type *LecRK* cluster expansion. We determined that several L-type *LecRK*s duplicated due to more ancient WGD events dating back to the time of divergence of the *A. thaliana* and *C. papaya* lineages approximately 72 Ma (Ming et al. 2008) and the divergence of *A. thaliana* and *V. vinifera* 111 Ma, respectively (supplementary table S5, Supplementary Material online). Our comparative analysis also showed evidence for the impact of GTD to L-type *LecRK* gene copy number and divergence. All ambiguous L-type *LecRK*s, that is, those that do not belong to a distinct clade (fig. 2*A*), showed evidence for GTD, which was confirmed in our phylogenetic analysis (fig. 2*B*). Subjection of genes to GTD may also result into fractionation of gene collinearity, thereby introducing target genes to a novel genomic context and thus influencing functional divergence across L-type *LecRK* clades or even genomes.

Here, we demonstrate that L-type *LecRK*s have undergone all modes of duplication in their evolutionary history, with the highest fraction of duplicates due to WGD and TD. Recent WGD and TD have by far most influenced the birth of L-type *LecRK*s and might be a factor for their functional divergence. L-type *LecRK*s form a family whose stability is manifested in the syntenic retention across Brassicaceae species and other closely related species. Earlier findings showed that different duplication events occurred at different times during evolution (Jaillon et al. 2007; Ming et al. 2008; Wang et al. 2009; Woodhouse et al. 2011; Schranz et al. 2012; Vekemans et al. 2012; Cheng et al. 2013). However, our results demonstrate an exceptional simultaneous occurrence of WGD and TD for L-type *LecRK*s across species. This makes the L-type *LecRK* family a highly dynamic and interesting exception among several other studied gene families (Hofberger et al. 2013, 2014). We also established that *LLP*s cluster into two clades based on sequence homology. It is likely that their origin is due to domain loss from L-type LecRK proteins. Hence, LLPs likely acquired novel functions; however, future functional analysis is important to confirm this hypothesis. In this study, we propose a uniform nomenclature for the *A. thaliana* LLPs based upon two criteria: 1) Clustering in the phylogenetic tree with PP values greater than 0.9, and 2) the presence or absence of a transmembrane domain (i.e., the LecRPs vs. LecPs) (fig. 1). This was inspired by the nomenclature given to the L-type LecRKs by Bouwmeester and Govers (2009). LLPs share an evolutionary history with L-type LecRKs based on synteny and the monophyletic grouping with specific L-type *LecRK* clades.

Overall, our findings reveal a dynamic evolutionary history of genes encoding a legume-like lectin domain. This divergence is attributed to a complex interplay of WGD and TD events, thus resulting into domain retention and/or loss with subsequent sub- or neofunctionalization. We believe that the highly dynamic birth–death and expansion of these genes have contributed to plant immunity.

## Supplementary Material

Supplementary tables S1–S6 are available at *Genome Biology and Evolution* online (http://www.gbe.oxfordjournals.org/).

Supplementary Data
