# Supplementary material for: A Complex Interplay of Tandem- and Whole-Genome Duplication Drives Expansion of the L-Type Lectin Receptor Kinase Gene Family in the Brassicaceae
Source: Genome Biol Evol. 2015 Jan 28;7(3):720–34. doi: 10.1093/gbe/evv020 (PMC5322546; doi:10.1093/gbe/evv020)
Supplement: Supplementary Data [file supp_7_3_720__index.html]

A Complex Interplay of Tandem- and Whole-Genome Duplication Drives Expansion of the L-Type Lectin Receptor Kinase Gene Family in the Brassicaceae — Supplementary Data 

# A Complex Interplay of Tandem- and Whole-Genome Duplication Drives Expansion of the L-Type Lectin Receptor Kinase Gene Family in the Brassicaceae

## Supplementary Data

files

**Files in this Data Supplement:**

- Supplementary Data - docx file
- Supplementary Data - xlsx file
- Supplementary Data - xlsx file
- Supplementary Data - xlsx file
- Supplementary Data - xlsx file
- Supplementary Data - xlsx file
- Supplementary Data - xlsx file
