# Supplementary material for: A Complex Interplay of Tandem- and Whole-Genome Duplication Drives Expansion of the L-Type Lectin Receptor Kinase Gene Family in the Brassicaceae
Source: Genome Biol Evol. 2015 Jan 28;7(3):720–34. doi: 10.1093/gbe/evv020 (PMC5322546; doi:10.1093/gbe/evv020)
Supplement: Supplementary Data [file supp_evv020_New_Microsoft_Office_Word_Document.docx]

**Supplementary Table I**. All identified genes encoding a legume-like lectin domain and modes of duplication across nine analysed genome assemblies

**Supplementary Table II**. Ohnologs to *A. thaliana* L-type *LecRK* genes across eight target genomes

**Supplementary Table III**. Orthologs to *A. thaliana* L-type *LecRK* genes across eight target genomes

**Supplementary Table IV**. Orthologs to *A. thaliana LLP* genes across eight target genomes

**Supplementary Table V**. Ohnologs to *A. thaliana LLP* genes across eight target genomes

**Supplementary Table VI**. Duplicates among *A. thaliana* L-type *LecRK* genes and duplication mode
